# Hot Gas Pressure Forming of Ti-55 High Temperature Titanium Alloy Tubular Component

**DOI:** 10.3390/ma13204636

**Published:** 2020-10-17

**Authors:** Kehuan Wang, Chenyu Shi, Shiqiang Zhu, Yongming Wang, Jintao Shi, Gang Liu

**Affiliations:** 1 National Key Laboratory for Precision Hot Processing of Metals, Harbin Institute of Technology, Harbin 150001, China; shichenyu123@foxmail.com (C.S.); hitzsq@126.com (S.Z.); gliu@hit.edu.cn (G.L.); 2 Institute of High Pressure Fluid Forming, Harbin Institute of Technology, Harbin 150001, China; 3 Capital Aerospace Machinery Corporation Limited, Beijing 100076, China; wangyongming211@163.com (Y.W.); 13801337357@139.com (J.S.)

**Keywords:** Ti-55 titanium alloy, hot gas pressure forming, processing windows, microstructure, post-form strength

## Abstract

In this paper, hot gas pressure forming (HGPF) of Ti-55 high temperature titanium alloy was studied. The hot deformation behavior was studied by uniaxial tensile tests at temperatures ranging from 750 to 900 °C with strain rates ranging from 0.001 to 0.05 s^−1^, and the microstructure evolution during tensile tests was characterized by electron backscatter diffraction. Finite element (FE) simulation of HGPF was carried out to study the effect of axial feeding on thickness distribution. Forming tests were performed to validate this process for Ti-55 alloy. Results show that when the temperature was higher than 750 °C, the elongation was large enough for HGPF of Ti-55 alloy. Dynamic recrystallization (DRX) occurred during the tensile deformation, which could refine the microstructure. The thickness uniformity of the formed part could be improved by increasing feeding length. The maximum thinning ratio decreased from 27.7% to 11.5% with the feeding length increasing from 0 to 20 mm. A qualified Ti-55 alloy component was successfully formed at 850 °C, the microstructure was slightly refined after forming, and the average post-form yield strength and peak strength were increased by 8.7% and 6.9%, respectively. Pre-heat treatment at 950 °C before HGPF could obtain Ti-55 alloy tubular component with bimodal microstructure and further improve the post-form strength.

## 1. Introduction

Complex thin-walled components made of titanium alloys are always very popular in the aviation and aerospace industries due to their excellent comprehensive mechanical properties and the pronounced effect in reducing weight [1,2]. With the rapid development of high-speed vehicles, the temperatures of some components such as compressor discs, blades, and panel parts could exceed 500 °C [3,4]. In order to reduce weight and increase the flexibility of the vehicle, high-temperature titanium alloys such as IMI834, IMI829, Ti1100, Ti-55, Ti-60, and Ti-65 are very competitive candidates for those components compared with steels or nickel alloys [3,5,6,7]. However, determining how to manufacture complex thin-walled components made of high-temperature titanium alloys efficiently is difficult because of the high deformation resistance and severe springback at room temperature [8]. Complex thin-walled titanium alloys components were traditionally formed by superplastic forming [9]. However, both the high forming temperature and long forming time not only increased the cost greatly but also impaired the post-form properties [10], which limited its wide application. With regards to the forming of titanium alloys tubular components, they were usually formed by hot pressing and welding, namely forming two halves first and then welding them together. In order to improve the forming efficiency and reliability of titanium alloys tubular components, hot gas pressure forming (HGPF) for titanium alloys was developed [8]. However, most of the studies focused on the forming of low-strength titanium alloys such as TA2 [11], grade 2 commercially pure titanium [12], and Ti-3Al-2.5V titanium alloy [13,14,15].

Ti-55 alloy with a nominal composition of Ti-5Al-4Sn-2Zr-1Mo-0.25Si-1Nd (wt.%) is a near-α high-temperature titanium alloy which was designed to serve at 550 °C. Lots of fundamental researches about this alloy have been reported. The activation energies of Ti-55 alloy in the two-phase and single-phase regions were calculated to be 453 and 279.88 KJ/mol, respectively, by Wu [16], and the main softening mechanism during hot deformation is dynamic recrystallization of α phase and dynamic recovery of *β* phase [16]. The recrystallization of Ti-55 alloy during the hot compression process was simulated by cellular automaton in [17]; it was found that temperature and strain rate affect recrystallization a lot and most of the recrystallized grains nucleate at the deformed grain boundaries. Liu [18] revealed that the main deformation mechanism of Ti-55 alloy at temperatures ranging from 885 °C to 935 °C is grain boundary sliding accommodated with grain rotation, which is similar with the superplastic deformation of two-phase titanium alloys. Wu [19] found that 850 °C is a proper temperature for the hot press forming of Ti-55 alloy and a complex curvilinear generatrix workpiece without wrinkles is formed by two-step hot press forming process. Because of the higher strength at elevated temperature, the temperature for hot press forming of Ti-55 alloy is about 150 °C higher than that for TC4 alloy. Liu [20] studied the superplastic deformation behavior of Ti-55 alloy by uniaxial tensile tests and found that a maximum elongation of 987% could be achieved at 925 °C with strain rate of 6.64 × 10^−3^ s^−1^. In order to reduce the forming temperature, hydrogenation treatment of Ti-55 alloy was performed by Li [21].The optimum superplastic temperature of the hydrogenated Ti-55 alloy could be reduced by about 100 °C compared with the un-hydrogenated one because of higher volume fraction of *β* phase, appropriate *α/β* phase ratio, grain refinement, and dynamic recrystallization [21,22]. Most of the researches about Ti-55 alloy focused on the relation between the microstructure and properties, hot pressing, and superplastic forming. Few researches on HGPF could be found about high-temperature titanium alloys. Effects of forming parameters on microstructure evolution and post-form strength are still unclear.

In this paper, the hot deformation behavior and microstructure evolution of Ti-55 alloy under HGPF condition were studied to determine proper processing windows for HGPF. FE simulation of HGPF was adopted to study the effect of axial feeding on thickness distribution. A Ti-55 alloy tubular component with a large diameter variance was successfully formed by HGPF, and pre-heat treatment was also employed to tailor the post-form microstructure and properties.

## 2. Materials and Methods

### 2.1. Materials

**Figure 1:**
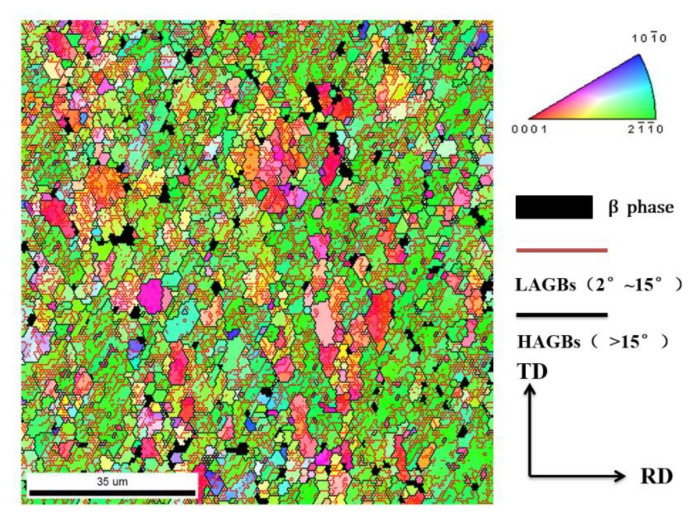
Inverse polar figure (IPF) of the as-received Ti-55 alloy.

A Ti-55 alloy hot rolled sheet with an average thickness of 1 mm was used in this paper. The initial microstructure of the as-received sheet is shown in Figure 1 by inverse polar figure (IPF).

The as-received material had an equiaxed microstructure with an average grain size of 2.5 μm. The grain size varied from 0.3 to 30 μm, with small grains distributed along the boundaries of large grains, indicating that recrystallization occurred during the rolling or annealing process. The deformed grains were full of low angel grain boundaries (LAGBs) inside, which was inherited from the previous rolling deformation. The chemical composition (in wt.%) of the as-received sheet is shown in Table 1.

### 2.2. Hot Gas Pressure Forming

**Figure 2:**
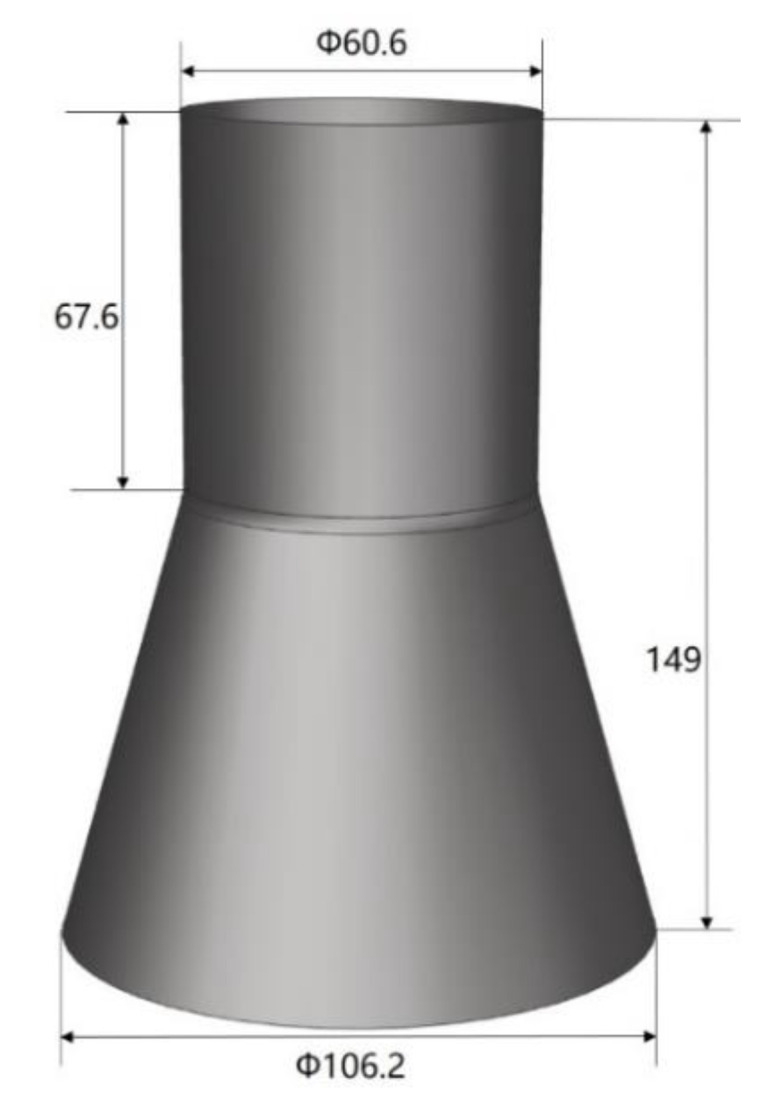
Geometry of Ti-55 alloy tubular component with a large diameter variance.

A Ti-55 alloy tubular component with a large diameter variance was formed by HGPF. The geometry of the component is shown in Figure 2.

**Figure 3:**
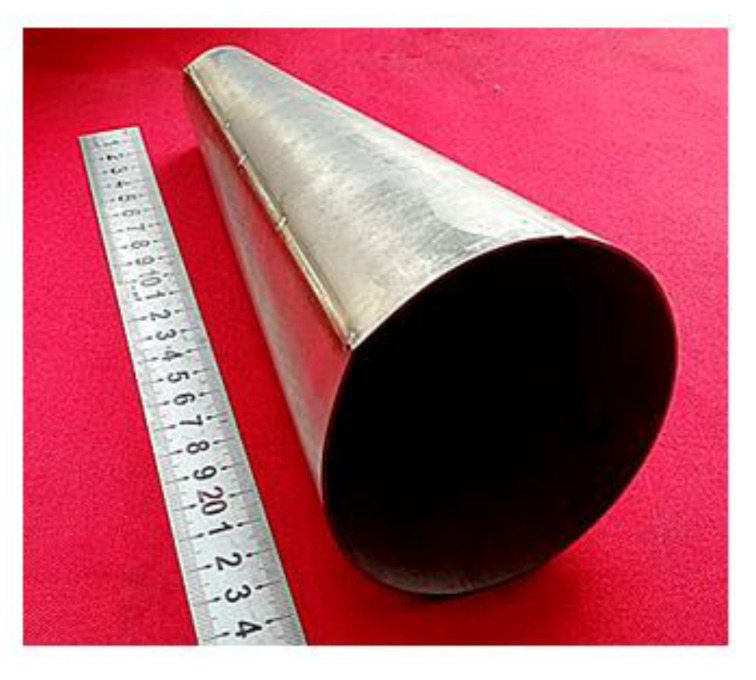
The initial cone tube made by U-O forming and laser beam welding.

The component consists of two sections. One section is a cylinder with a diameter of 60.6 mm, and the other section is a cone with the largest diameter of 106.2 mm, which is 1.75 times of the smallest diameter. If this component was formed directly with a straight tube, severe thinning would occur even with the axial feeding. Therefore, a cone tube was designed to reduce the expansion ratio and improve the thickness uniformity. The initial cone tube was fabricated by U-O forming and laser beam welding as shown in Figure 3, and more details about U-O forming could be found in [23].

According to our previous study of laser beam welding of a near-α titanium alloy [24], the weld zone has a microstructure consisting of coarse columnar grains with fine *α*′ martensitic structure in the matrix because of the rapid cooling from the melting temperature. The weld seam has a higher strength but lower ductility than the parent material at both room and elevated temperature. The weld seam would hinder the deformation of the tube a bit during the HGPF due to its higher strength, but the weld seam is too narrow in width to have an obvious effect on the uniformity of the deformation before the occurrence of plastic instability [23]. The maximum expansion ratio of the cone tube in this study was only 32.2%. Therefore, the effect of the weld seam on the deformation was neglected in this paper.

**Figure 4:**
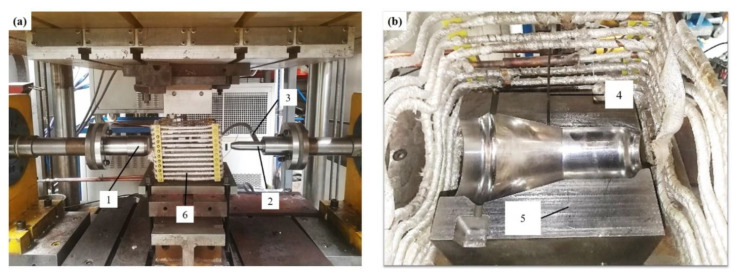
Forming apparatuses for hot gas pressure forming (HGPF) of Ti-55 alloy, where 1 is left punch, 2 is right punch, 3 is gas inlet, 4 is thermocouple, 5 is the lower die, and 6 is the induction coils. (**a**) The overall setup and (**b**) the lower die.

The forming apparatus for HGPF of Ti-55 alloy is shown in Figure 4, which includes forming dies, seal punches, induction heating device, high pressure gas source, heat insulation, and press cooling modules.

During the forming, the forming dies were heated to the targeted temperature by induction coils firstly, and then the tube was placed into the lower die soaking for 10 min before forming. The temperature of the tube was detected by a thermocouple. After the soaking, the heated tube was sealed by two punches, and then the compressed gas and axial feeding were loaded simultaneously according to the designed loading path. The formed tube was taken out and quenched in water immediately after the forming.

### 2.3. Uniaxial Tensile Tests and Microstructure Characterization

Uniaxial tensile tests at temperatures ranging from 750 to 900 °C with strain rates ranging from 0.001 to 0.05 s^−1^ were carried out to study the hot deformation behavior of Ti-55 alloy and determine the processing windows for HGPF. The samples for uniaxial tensile tests were machined from the as-received sheet along the rolling direction. The gauge length of the sample was 15 mm, and the width was 5 mm. All the tensile tests were performed at least twice to guarantee the repeatability. The stretched samples were quenched in water immediately after the test to freeze the microstructure at elevated temperature.

The effects of processing parameters including temperature and strain rate on the microstructure were investigated by electron backscatter diffraction (EBSD). After the tensile tests, samples for microstructure characterization were taken from the deformed sample near the fracture area. The EBSD was performed on a Zeiss Supra55 scanning electron microscope operated at 20 kV with a step size of 0.2 µm. The samples for EBSD measurement were prepared by electro-polishing with a solution of 6% perchloric acid, 34% butanol, and 60% methanol (vol %) at −40°C with a potential of 30 V and current of 0.8 A.

## 3. Results and Discussion

### 3.1. Hot Deformation Under Uniaxial Tensile Condition

**Figure 5:**
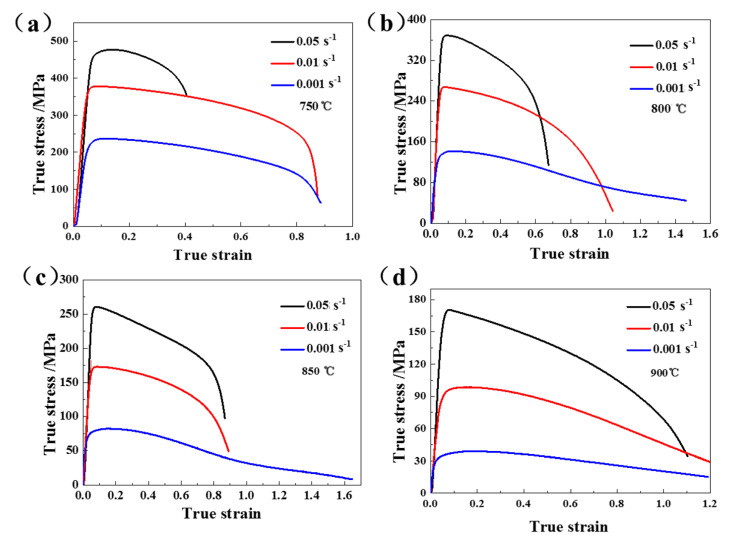
Stress–strain curves of Ti-55 alloy under different conditions (**a**) 750 °C; (**b**) 800 °C; (**c**) 850 °C; and (**d**) 900 °C.

The true stress–true strain curves of Ti-55 alloys are shown in Figure 5.

**Figure 6:**
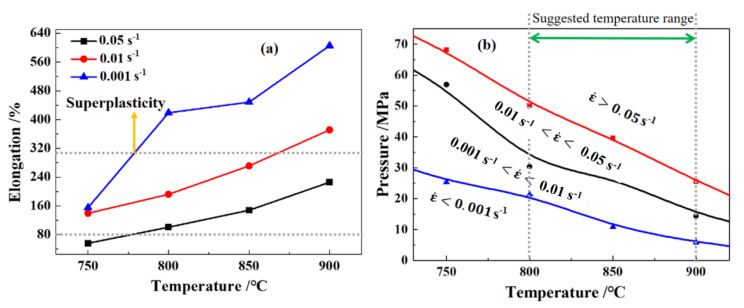
Elongation distribution (**a**) and forming pressure (**b**) of Ti-55 alloy under different conditions.

The flow stress decreased with the increasing temperature and decreasing strain rate, which is typical for titanium alloys. Obvious material softening occurred at all of the test conditions. Compared with another widely used near-α titanium alloy, namely TA15, the flow stress of Ti-55 alloy was about 100 MPa higher and the strain-to-failure of Ti-55 alloy was about 0.2 lower, indicating that it is more challenging to form components made of Ti-55 alloy. The processing windows for HGPF of Ti-55 alloy should be built according to the elongation, forming pressure, forming temperature, post-form microstructure, and property. The elongation distribution of Ti-55 alloy at different temperatures and strain rates is shown in Figure 6a.

When the temperature was higher than 750 °C, the elongation was higher than 80% under all the tests conditions, and superplasticity was observed as the strain rate was lower than 0.01 s^−1^. Therefore, the elongation is pretty enough for HGPF of Ti-55 alloy, when the temperature was higher than 750 °C.

The forming pressure for HGPF of Ti-55 alloy could be estimated according to the flow stress and the geometry of the component as shown by Equation (1) [25]:

(1) $$p=\frac{t}{r}\sigma_{p}$$

where p is the forming pressure, t is the average thickness, r is the radius of the smallest corner of the component, and $\sigma_{p}$ is the peak stress of the material. The average thickness of Ti-55 alloy sheets used in this paper was 1 mm, the r value was set as 6 mm according to geometry of the component, and $\sigma_{p}$ was obtained from Figure 5. The calculated results are shown in Figure 6b. The forming pressure needed at strain rate of 0.001 s^−1^ and temperature of 800 °C was about 25 MPa, which is much higher than the pressure in the standardized argon gas cylinder. Hence, the forming temperature for HGPF of Ti-55 alloy is suggested to be no less than 800 °C. The forming pressure increased with the strain rate. As the strain rate was 0.01 s^−1^, the forming pressure needed to reach this strain rate was higher than 30 MPa. Higher strain rate could improve the forming efficiency, but it will also increase the requirement of the forming apparatus. Therefore, the strain rate should be selected according to the capacity of the forming apparatus.

**Figure 7:**
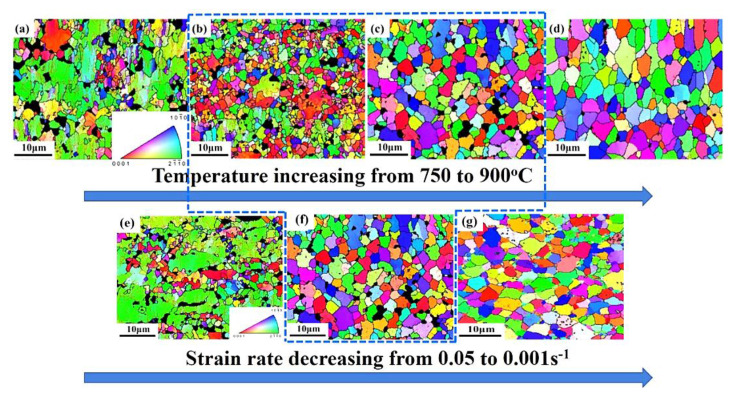
IPF figures of the samples after tensile deformation at (**a**) 750 °C, (**b**) 800 °C, (**c**) 850 °C, and (**d**) 900 °C with strain rate of 0.01 s^−1^, and with strain rate of (**e**) 0.1 s^−1^, (**f**) 0.01 s^−1^, and (**g**) 0.001 s^−1^ at 850 °C (the black color represents *β* phase).

The microstructures of Ti-55 alloy after tensile deformation at different temperatures and strain rates are shown in Figure 7.

The average grain size decreased first, and then increased with the increasing temperature under the same strain rate condition as shown in Figure 7a–d. The average grain size increased with the decreasing strain rate at 850 °C as shown in Figure 7e–g. This is because dynamic recrystallization (DRX) occurred. During the hot deformation of Ti-55 alloy, the microstructure evolution was mainly determined by recovery, recrystallization or phase transformation [26]. Recovery had little effect on grain size. Phase transformation at 800 °C was not obvious because the β transformation temperature of Ti-55 alloy was as high as 995 °C. Therefore, DRX was the dominating reason for the grain refining. The sample after deformation at 800 °C had the smallest average grain size (Figure 7b). But when the temperature reached 900 °C, the recrystallized grains grew larger with the increasing strain. This is because DRX nucleation and grain growth occurred simultaneously during the deformation. Higher temperature accelerated both nucleation and grain growth [27]; therefore, full DRX could be accomplished sooner at higher temperature and the further deformation would lead to grain growth [27]. At the same temperature, more DRX occurred with the decreasing strain rate. As strain rate was decreased to 0.001 s^−1^, grain growth was also observed. However, the uniformity of the grain size distribution was terrible under low temperature (Figure 7a) and high strain rate conditions (Figure 7e) because of the partial recrystallization, where recrystallized fine grains distributed along the grain boundaries of the large deformed grains, indicating that the main DRX mechanism was discontinuous DRX. Therefore, temperatures ranging from 800 to 850 °C with strain rate ranging from 0.01 to 0.001 s^−1^ was recommended for the HGPF of Ti-55 alloy in the perspective of microstructure.

### 3.2. Effect of Axial Feeding on Thickness Distribution

The forming temperature and strain rate affect formability and post-form properties of Ti-55 alloy component. However, the thickness accuracy was mainly controlled by expansion ratio and axial feeding. The dimension of the initial cone tube was determined according to the geometry characteristic of the component with the aim to reduce the overall expansion ratio. The diameters of the initial cone at the two ends are 35.7 and 92.8 mm, respectively, and the length is 228.1 mm. The maximum expansion ratio at the left and right side are 24.5% and 32.2%, respectively.

**Figure 8:**
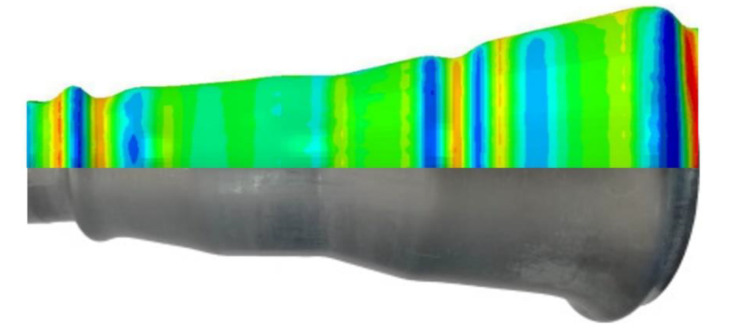
Comparison of the simulation and experimental results.

A FE model of HGPF of Ti-55 alloy tubular component was built in Abaqus to investigate the effect of axial feeding on the thickness distribution. Stress–strain curves at 850 °C as shown in Figure 5 were input to fit the constitutive equation. The forming dies and punches were defined as rigid bodies and the tube was assigned as deformable shell element. During the simulation, all degrees of freedom of the lower die was fixed, the upper die moved vertically until contacting the lower die, and the two punches move horizontally according to the loading paths. The Coulomb friction model was used for all contact surfaces with the coefficient of 0.1. The simulation was validated by comparing the simulation result with the experimental result as shown in Figure 8.

The position and shape of the wrinkles in the simulation matched well with the experimental result, indicating the good reliability of the simulation.

**Figure 9:**
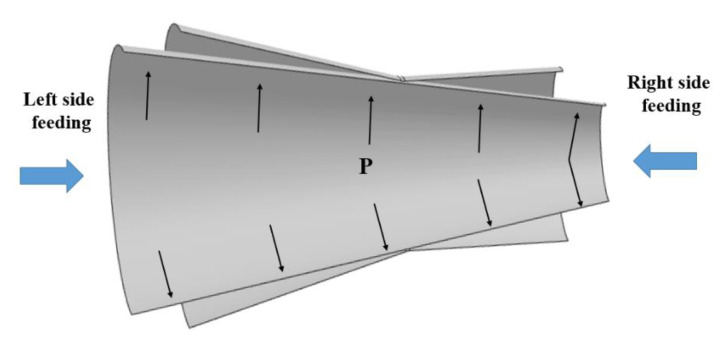
Schematic figure of the relationship between the initial cone tube, the final component, and axial feeding.

Because a cone tube was used in the HGPF, the axial feeding at different sides (Figure 9) would affect the thickness distribution.

**Figure 10:**
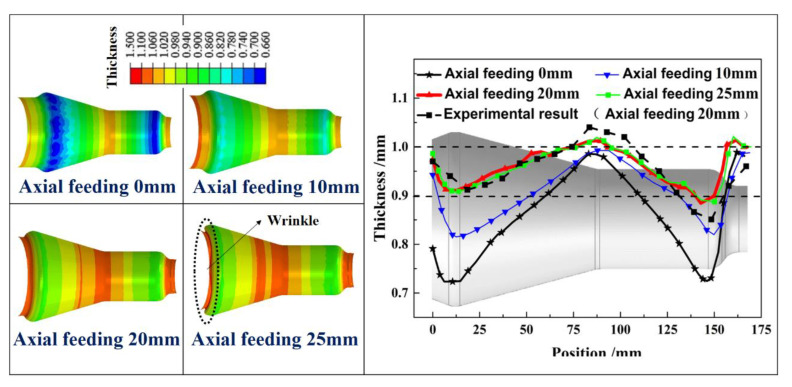
Effect of axial feeding on thickness distribution.

For example, if the axial feeding was performed at the right side, the initial expansion ratio would increase and therefore led to more material thinning. Hence, four FE simulations with axial feeding length of 0, 10, 20, and 25 mm at the left side were performed and the thickness distribution result is shown in Figure 10.

The experimental result with axial feeding of 20 mm was also provided, from which one can tell that the simulation results agreed well with the experimental results. When the axial feeding length was 0 mm, severe thinning occurred near the two corner areas at the two sides because of the large expansion at the two corners according to Figure 9. The thinning ratio decreased gradually from the two sides to the middle area due to the decreasing expansion ratio. The maximum thinning occurred at the corner area of the right or left side, and it decreased from 27.7% to 11.3% with the feeding length increasing from 0 to 25 mm. The thickness uniformity of the formed part was improved with the increasing feeding length. However, the thickness distribution changed little when the feeding length was higher than 20 mm, and wrinkle occurred at the left side when the feeding length was 25 mm. This is because when the axial feeding reached 20 mm, most of the formed part contacted with the forming tools, and it would be very difficult to feed more material into the die cavity due to the pronounced friction stress. Therefore, axial feeding after 20 mm could not improve the thickness uniformity but resulted in wrinkling at the left side.

### 3.3. HGPF of the Ti-55 Alloy Component

**Figure 11:**
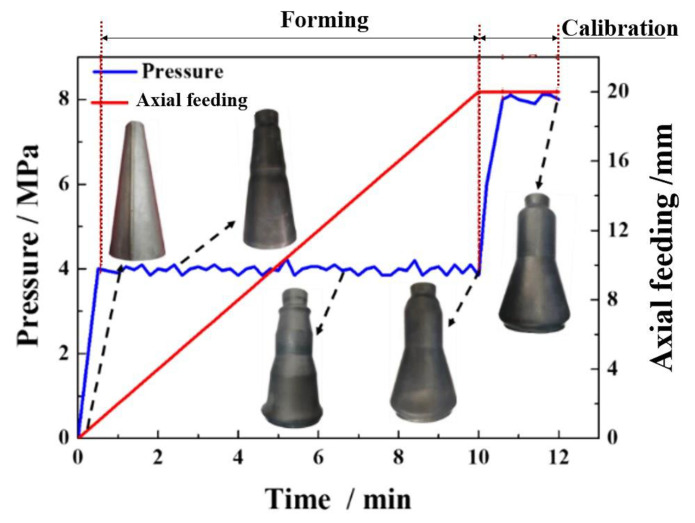
Loading path of the HGPF of the Ti-55 alloy component at 850 °C.

The HGPF of the Ti-55 alloy component was performed at 850 °C according to the above studies, and the loading paths including gas pressure and axial feeding are shown in Figure 11.

**Figure 12:**
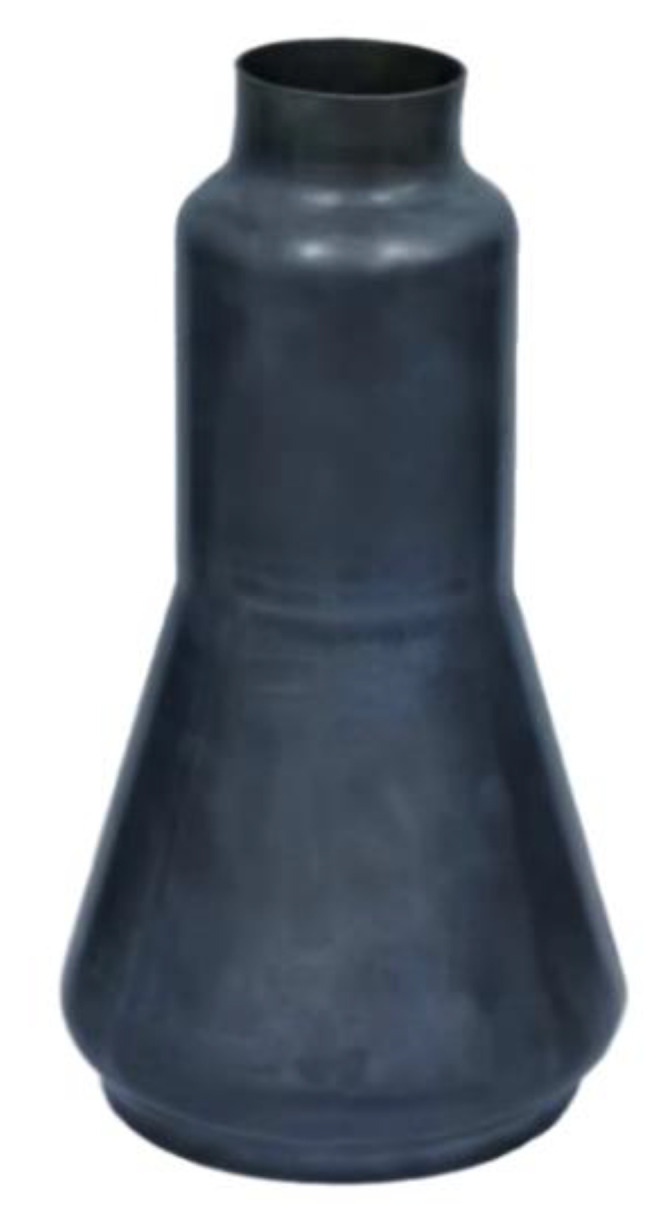
The final formed Ti-55 alloy component by HGPF.

The gas pressure was increased to 4 MPa firstly and was kept nearly constant during the sequent forming, and meanwhile the axial feeding at the left side was performed until the feeding length reached 20 mm according to the simulation results. At the forming time of about 2 min, a small “useful wrinkle” appeared at the small end of the tube, and the wrinkle became obvious at both ends with the increasing of feeding length. At the time of about 10 min, most of the tube reached the designed position under the conjunction function of axial feeding and gas pressure. Then, the formed part was calibrated by increasing the pressure to 8 MPa and held for 2 min. The final formed qualified part is shown in Figure 12.

The overall thickness distribution tendency of the formed part is the same with the simulation results (Figure 10). The maximum thinning occurred at the corner area of the small end and the thinning ratio is 14.9%, which is in good agreement with the simulation results.

### 3.4. Post-Form Microstructure and Properties of the Ti-55 Alloy 

**Figure 13:**
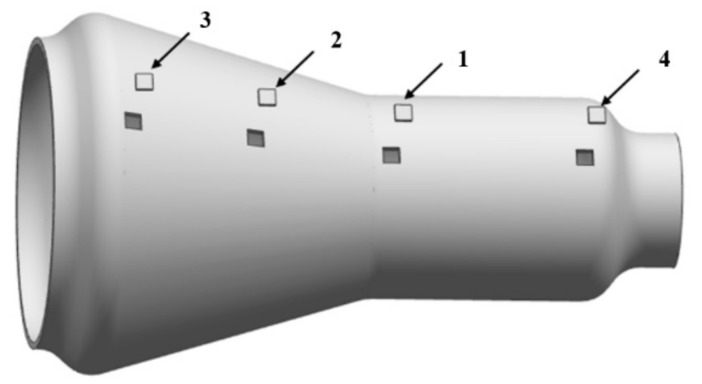
Samples positions and the corresponding equivalent strains (1) 0; (2) 0.12; (3) 0.22; and (4) 0.28.

To evaluate the effect of HGPF on the post-form microstructure and properties of the Ti-55 alloy, samples with different equivalent strains were taken from the formed part, and the positions of the samples are shown in Figure 13.

**Figure 14:**
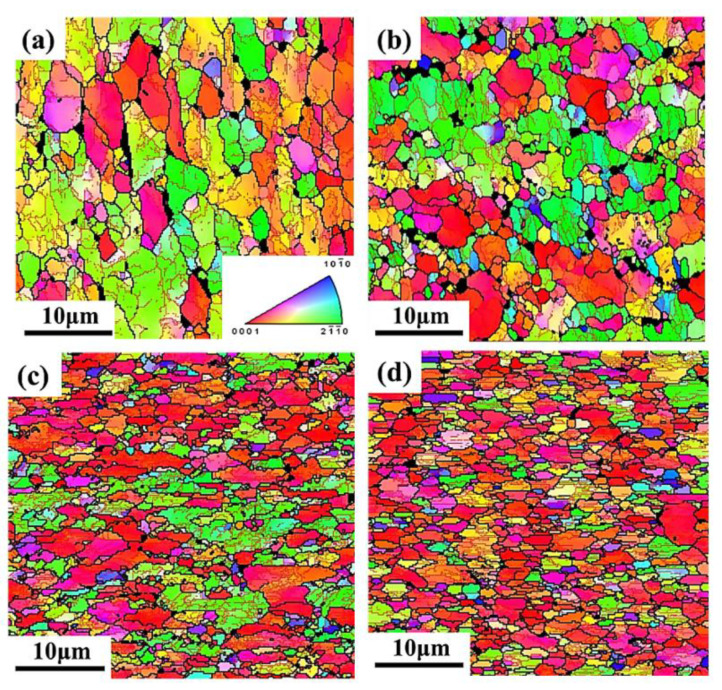
Microstructure of the final formed part in IPF figures with different equivalent strain of (**a**) 0; (**b**) 0.12; (**c**) 0.22; and (**d**) 0.28.

The equivalent strains of the four samples are approximate 0, 0.12, 0.22, and 0.28, respectively. The microstructures of the samples with different equivalent strain are shown in Figure 14 by IPF figures.

**Figure 15:**
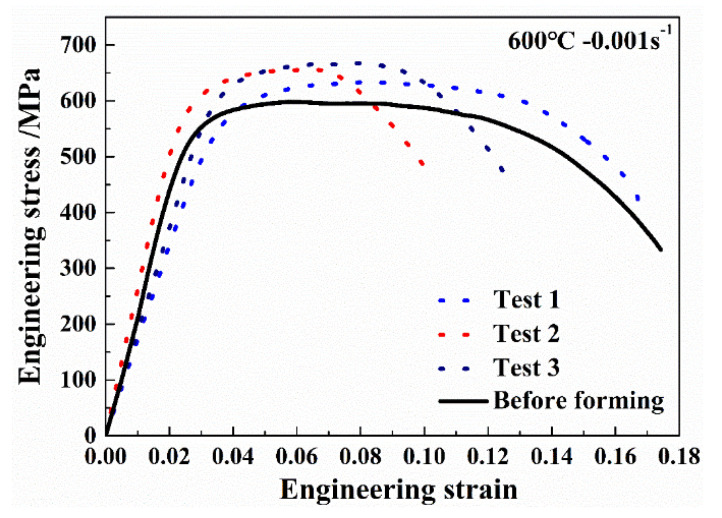
Engineering stress–strain curves of the Ti-55 alloy after HGPF.

The average grain size decreased with the increasing equivalent strain because of DRX. The average grain size of sample (1) with equivalent strain of 0 was 2.1 μm, which is 0.4 μm less than the as-received material, indicating that both forming temperature and period were appropriate for Ti-55 alloy to avoid grain growth, and static recrystallization also occurred during the forming, leading to the slight refinement of the grain size. When the equivalent strain was increased to 0.12, lots of fine recrystallized grains appeared along the grain boundaries after forming. The average grain size was further refined to 1.3 μm with equivalent strain increased to 0.28. During the HGPF of Ti-55 alloy, recrystallized grains firstly nucleated at the grain boundaries of the deformed grains, and with the increasing strain and forming time, more DRX occurred and some of the recrystallized grains grew up gradually. Therefore, the main DRX mechanism of Ti-55 alloy during HGPF would be discontinuous DRX according to the characteristic of the microstructure evolution. The service property of the final formed part was evaluated by uniaxial tensile tests at 600 °C and the results are shown in Figure 15.

The average yield stress and peak stress of Ti-55 alloy after HGPF were 564.2 and 642.1 MPa, respectively, which were about 8.7% and 6.9% higher than that before HGPF. However, the average elongation was reduced slightly after HGPF.

### 3.5. Adjustment of Microstructure of the Ti-55 Alloy by Pre-Heat Treatment

**Figure 16:**
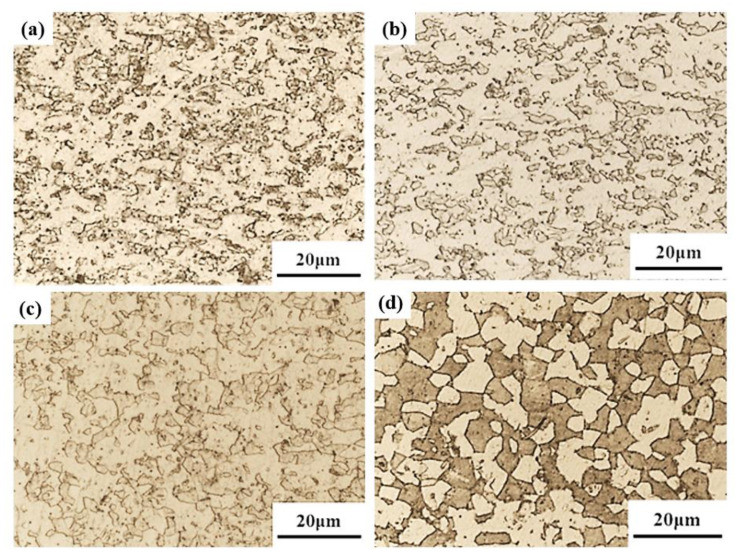
Microstructure of Ti-55 alloy sheet after annealing at (**a**) 800 (**b**) 850 (**c**) 900, and (**d**) 950°C for 2 h followed by water-quenching.

How to guarantee dimensional accuracy, microstructure, and mechanical properties simultaneously after plastic forming is a great challenge for titanium alloys because of the high deformation resistance and complex microstructure evolution at elevated temperature. Bimodal microstructure for titanium alloys is often favorable because of its good comprehensive mechanical properties. If one wants to achieve such a microstructure after HGPF, pre-heat treatment is necessary. In order to get the proper pre-heat treatment scheme, annealing at temperatures of 800, 850, 900, and 950 °C followed by water-quenching were performed. The optical microstructures after different annealing were shown in Figure 16.

Both volume fraction and size of *β* phase increased with the increasing temperature, especially when the temperature was higher than 850 °C. After the Ti-55 alloy was heat-treated at 950 °C, martensite or fine secondary α formed inside the *β* matrix, which could improve the post-heat treatment strength. In order to obtain the bimodal microstructure for Ti-55 alloy after HGPF, pre-heat treatment at 950 °C is suggested. 

**Figure 17:**
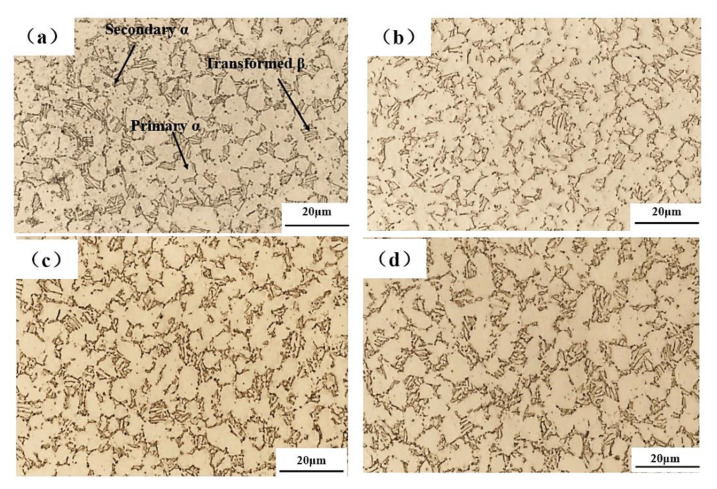
Microstructure of the annealed Ti-55 alloy tube after HGPF with different equivalent strain of (**a**) 0; (**b**) 0.12; (**c**) 0.22; and (**d**) 0.28.

Before the HGPF, pre-heat treatment at 950 °C for 2 h followed by water-quenching was performed according to the above results. The microstructures of the formed part with different equivalent strains are shown in Figure 17 by optical microscope figure, where the microstructures in different areas with different equivalent strains were similar including primary *α*, secondary *α*, and transformed *β*.

The fraction of transformed *β* phase was much less than that after heat treatment only and the secondary α became shorter and thicker. This is because transformed *β* phase decomposed to α and *β* during HGPF, and the globalization of secondary α also occurred during the HGPF. Therefore, fraction of primary α increased after HGPF.

**Figure 18:**
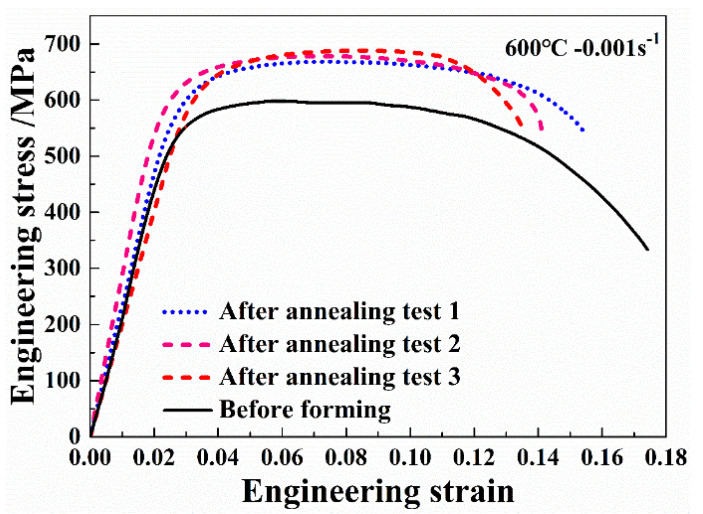
Engineering stress–strain curves of the annealed Ti-55 alloy after HGPF.

Uniaxial tensile tests at 600 °C were performed to evaluate the effect of pre-heat treatment on the service property of Ti-55 alloy and the results are shown in Figure 18.

Three samples were taken from the formed part and the three curves agreed well with each other, indicating that the post-form strength distributed uniformly over the formed part. Compared with the material before forming, the average yield strength and peak stress were increased by 12.0% and 12.9%, respectively, after HGPF with pre-heat treatment. However, the average elongation was reduced slightly after HGPF.

It could be seen from the above results that pre-heat treatment is an effective method to tailor the post-form microstructure and properties for HGPF of Ti-55 alloy, which could avoid the geometry distortion during the traditional heat treatment after forming. One could extend this solution to adjust Ti-55 alloy to other microstructures by changing the scheme of the pre-heat treatment according to the requirements of the specific product. 

## 4. Conclusions

In this paper, HGPF of a Ti-55 alloy tubular component with a large diameter variance was studied. The main conclusions could be drawn as follows:

1. When the temperature was higher than 750 °C, the elongation of Ti-55 alloy was higher than 80%, and superplasticity was observed as the strain rate was lower than 0.01 s^−1^. The forming pressure needed during HGPF increased with the decreasing temperature and increasing strain rate.
2. Thermomechanical parameters including temperature, strain, and strain rate affect the DRX of Ti-55 alloy a lot. More DRX would occur with the increasing temperature, strain, and decreasing strain rate. The average grain size decreased first and then increased with the increasing fraction of recrystallized grains. The main DRX mechanism was discontinuous dynamic recrystallization.
3. Temperatures ranging from 800 to 850 °C with strain rate ranging from 0.01 to 0.001 s^−1^ was recommended for the HGPF of Ti-55 alloy by considering the proper elongation, forming pressure, and post-form microstructure. Axial feeding of 20 mm at the big end was recommended because more feeding cannot improve the thickness uniformity anymore but resulted in wrinkling at the big end.
4. A qualified Ti-55 alloy tubular component with a large diameter variance was successfully formed at 850 °C with an initial cone tube, the microstructure was slightly refined after forming, and the average post-form yield stress and peak stress were increased by 8.7% and 6.9%, respectively. The main mechanism of the microstructure refinement during HGPF was also discontinuous dynamic recrystallization, which is the same with that under uniaxial tensile condition.
5. Pre-heat treatment of the initial tube is one effective method to tailor the post-form microstructure and properties of the Ti-55 alloy component formed by HGPF. After pre-heat treatment at 950 °C for 2 h followed by water-quenching, the final formed Ti-55 alloy component obtained a bimodal microstructure with the average yield strength and peak stress increased by 12.0% and 12.9%, respectively.

## Figures and Tables

**Table 1:** The chemical composition (wt.%) of the as-received Ti-55 alloy.

wt.%	Al	Sn	Zr	Mo	Nb	Si	Ta	Ti
Ti-55	5.4	3.3	2.9	1.0	0.4	0.3	0.4	Bal.
